# Age-related decline in temporal sound processing: insights from envelope steepness map in the mouse auditory cortex

**DOI:** 10.3389/fnagi.2026.1730629

**Published:** 2026-02-19

**Authors:** Tianrui Guo, Kuniyuki Takahashi, Shinsuke Ohshima, Tatsuya Yamagishi, Shuji Izumi, Chihiro Yagi, Ryota Kai, Akira Kimura, Hiroaki Tsukano, Arata Horii

**Affiliations:** 1 Department of Otolaryngology Head and Neck Surgery, Niigata University School of Medicine, Niigata, Japan; 2 Department of Otolaryngology Head and Neck Surgery, Faculty of Medicine, University of Miyazaki, Miyazaki, Japan; 3 Department of Psychiatry, University of North Carolina at Chapel Hill, Chapel Hill, NC, United States; 4 Neuroscience Center, University of North Carolina at Chapel Hill, Chapel Hill, NC, United States

**Keywords:** temporal sound processing, age-related decline, mouse auditory cortex, GCaMP6f, envelope steepness map

## Abstract

**Introduction:**

Spectral information of speech sounds is spatially arranged from low to high frequencies in the auditory cortex, known as tonotopic maps. Recently, we identified an envelope steepness map encoding the rise-ramp steepness of sounds, which is located orthogonally to the tonotopic map. Temporal components, particularly the sound envelope defined by the rise-ramp steepness, play a crucial role in speech perception. We hypothesized that the ability to sense temporal components of sounds, such as envelope steepness, would be diminished in aged mice.

**Methods:**

Responses to variations in rise-ramp time were investigated using transcranial macroscale calcium imaging of the auditory cortices of GCaMP6f-expressing mice. Normalized distance relative to the rise-ramp time of 0.01 ms was plotted for each rise-ramp time (0.1, 1, 10, and 100 ms) in the logarithmic scale graph: the steeper the slope of the fitted regression lines, the greater the distance between the peaks of the rise-ramp time of 0.01 ms and 100 ms. The slope of this regression line was compared between the different age groups: 1/3 months and 6/9/12 months after birth.

**Results:**

The slope of the fitted regression line for 5 kHz in the bilateral anterior auditory field and 20 kHz in the bilateral primary auditory field was significantly less steep in mice at 6/9/12 months after birth than at 1/3 months after birth.

**Conclusion:**

The shorter distance from the peak of 0.01 ms to 100 ms in animals 6/9/12 months after birth suggests difficulty in the separation of the rise-ramp time in aged animals. These findings may support the cortical mechanisms underlying age-related decline in speech perception.

## 1 Introduction

Speech sounds consist of two primary components, spectral and temporal. Spectral components, also known as carrier frequencies, generate pitch perception and contribute to the identification of intonation. In contrast, temporal components, particularly the sound envelope, which represents the dynamic fluctuation in sound amplitude over time (Drullman, 1995), play a crucial role in speech perception, especially in mediating voiced phonemes (Rosen, 1992; Shannon et al., 1995; Ríos-López et al., 2017; Aizawa and Eggermont, 2006). Because the temporal envelope is a conserved feature of vocalizations across mammalian species (Milne et al., 2016; Nittrouer et al., 2013; Aponte et al., 2021), the spectral–temporal dichotomy, particularly the temporal features, likely supports effective vocal communication across a broad range of mammalian species.

Regarding these two components, the spectral information is spatially arranged from low to high frequencies in the auditory cortex, known as tonotopic maps (Bizley et al., 2005). A recent study demonstrated that the pitch of sounds was perceived through activity at corresponding locations on a tonotopic map (Ceballo et al., 2019). Although various naming conventions exist for areas of the mouse auditory cortex, in this study, we refer to auditory areas according to the classification presented by Narayanan et al.: anterior auditory field (AAF), primary auditory field (AI), ventral auditory field (VAF), and secondary auditory field (AII), where distinct tonotopic gradients were identified (Narayanan et al., 2022). In contrast to spectral components, the representation of the steepness of the sound envelope, which shapes the temporal components of speech sounds in the auditory cortex, remains poorly understood. Recently, we reported that the AAF and AI exhibited a specialized organization in response to variations in the steepness of the rise-ramp, one of the temporal components of sound, which is orderly mapped orthogonally to the tonotopic gradient, forming a two-dimensional representation on the cortical surface, suggesting that temporal envelopes and spectral tonotopic features are independently processed in the mammalian auditory cortex (Takahashi et al., 2025).

Age-related declines in speech recognition can occur despite adjustments in sound pressure levels (Eisenberg et al., 1995; Peters et al., 1998; George et al., 2006; Summers and Molis, 2004). The rise-ramp of the sound envelope may affect speech perception: sounds with a steep rise in the temporal envelope are easier to discriminate than those with a slow rise (Schlittenlacher and Ellermeier, 2015; Shimokura and Soeta, 2022). Given the important role of the temporal components of sounds in speech perception, age-related decline in speech perception may be attributed to a diminished ability to sense the temporal components of sounds, such as the rise-ramp. To gain deeper insight into the mechanisms underlying age-related decline in speech perception, we aimed to analyze how the auditory cortex responds to variations in the steepness of the rise-ramp across different ages using transcranial macroscale calcium imaging in the auditory cortices of GCaMP6f-expressing mice (Issa et al., 2014; Calhoun et al., 2023).

## 2 Methods

This study was approved by the Animal Care Committee of Niigata University School of Medicine (# SA00429 and # SD01079). All the experiments were performed in accordance with the approved guidelines and regulations.

### 2.1 Animals

We used 4–54-week-old Thy1-GCaMP6f mice (C57BL/6 J-Tg [Thy1-GCaMP6f] GP5.5Dkim/J) purchased from Jackson Laboratories. The animals were housed in cages with ad libitum access to food pellets and water and maintained on a 12-h light/dark cycle. Ten animals were used in each age group (1, 3, 6, 9, and 12 months after birth). The C57BL/6 J mice used in this study are known as a model for age-related hearing loss. Many previous reports have shown that age-related changes in high-frequency hearing can already be observed around 3 months of age, with further progression occurring at 6 and 9 months, and severe age-related hearing loss typically evident by 12 months (Harding et al., 2005; Yasuda et al., 2020; Someya et al., 2009; Tsukano et al., 2013). For this reason, although 6 months seem to be slightly early, we used it as a boundary to classify the animals into young and aged groups. Mice older than 12 months were not used, as they are highly likely to exhibit profound hearing loss at those ages.

### 2.2 Surgery

The mice were deeply anesthetized using urethane (1.35–1.65 g/kg, i.p.; Wako, Osaka, Japan) with atropine (0.5 mg/kg, s.c., Terumo, Tokyo, Japan). No additional anesthesia was administered. Typically, the surgery duration is within one hour, and duration of the measurements does not exceed five hours. After local anesthesia with 0.5% xylocaine (Aspen Pharmacare, Durban, South Africa), the skin and temporal muscles over the auditory cortex were incised, leaving the skull intact. A piece of metal was attached to the skull using dental resin, and the head was fixed by screwing the metal piece onto a manipulator. The exposed skull surface was covered with liquid paraffin (Wako, Osaka, Japan) to maintain transparency during imaging. The rectal temperature was maintained at 37 °C during surgery using a heat pad throughout the experiment.

### 2.3 Calcium imaging

**Figure 1:**
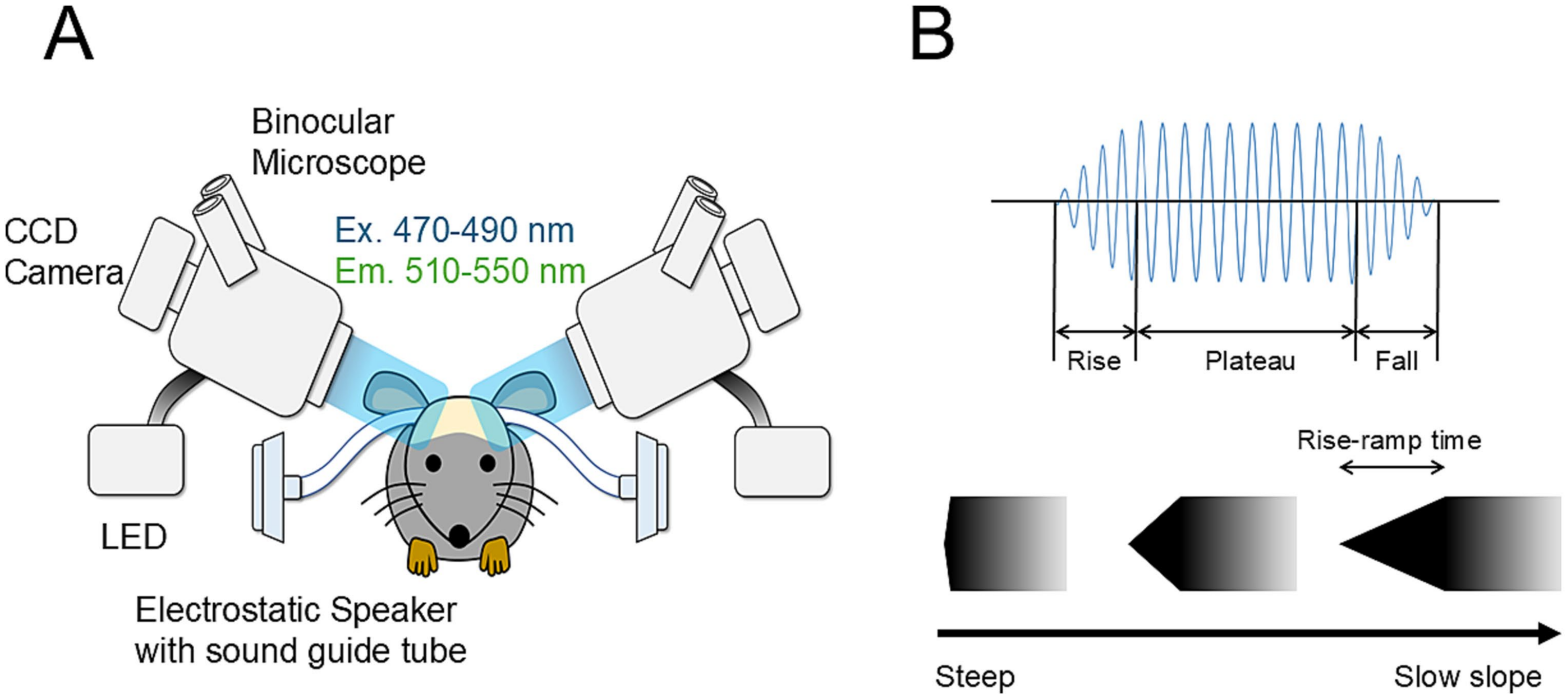
Schematic image of transcranial calcium imaging **(A)** and auditory stimuli **(B)**. **(A)** Macroscale transcranial calcium imaging of the bilateral auditory cortex was performed using GCaMP6f-expressing mice. Images were recorded using a CCD camera mounted on a microscope, which is controlled by RatioImaging Recorder. Fluorescence was excited at 470–490 nm and collected at 510–550 nm. Auditory stimuli were simultaneously presented to both ear canals via sound guide tubes connected to speakers. **(B)** Each stimulus had rise/fall times ranging from 0.01 to 100 ms corresponding to steep and slow slopes, respectively.

Approximately 30 min after the completion of surgery, macroscale calcium imaging was transcranially performed to visualize and identify functional activity maps in the bilateral auditory cortex (Figure 1A). Cortical images (128 × 168 pixels) were recorded using a cooled CCD camera system (ORCA-R2; Hamamatsu Photonics, Hamamatsu, Japan) attached to a microscope (M165FC, Leica Microsystems, Tokyo, Japan), which was controlled using a LabVIEW-based system (Ratio Imaging Recorder, E.I.SOL, Tokyo, Japan). The fluorescence of GCaMP6f, an indicator of neural activity, was excited using LED light (*λ* = 470–490 nm; UHP-Mic-LED-460, Opto-Line, Warabi, Japan). The emitted fluorescence was subsequently captured and filtered through bandpass filters (λ 510–550 nm).

Image analyses were performed using a LabVIEW-based system, RatioImaging Analyzer (E.I.SOL, Tokyo, Japan). Images were captured at a sampling rate of 20 Hz and averaged across 30 trials for each stimulus condition. Fluorescence changes were expressed as a pseudocolor scale in terms of the relative fluorescent change (ΔF/F0), where the baseline fluorescence intensity (F0) was calculated by averaging intensity values during a 250-ms pre-stimulus period. A spatial averaging of 5 × 5 pixels was applied. The locations of pixels with the maximum responses were identified by visual inspection in serial pseudocolor images.

### 2.4 Auditory stimuli

Sound waveforms were generated using the application (RPvdsEx) of a sound generator (System3; Tucker-Davis Technologies, Alachua, FL, United States) at a sampling rate of 130 kHz and were low-pass filtered at 100 kHz (3,625, NF, Kanagawa, Japan). Each stimulus had a duration of 1 s, with rise/fall times ranging from 0.01 to 100 ms (Figure 1B) and carrier frequencies of 5–40 kHz. Auditory stimuli were simultaneously delivered to both ear canals through dedicated sound guide tubes connected to speakers (EC1; Tucker-Davis Technologies). The sound intensity was set at approximately 60 dB SPL at the ear canal location. The sound intensity was calibrated using a sound level meter (NA-42S, RION, Kokubunji, Japan) equipped with a microphone and a preamplifier (UC54 and NH-17, RION). In this study, we fixed the sound level at 60 dB SPL for map visualization for two reasons. First, our work builds on the envelope steepness map that we previously identified (Takahashi et al., 2025). Because the angle of the envelope itself would change with sound pressure level (see Figure 1B), varying the sound level would compromise the consistency of data trends and their interpretation unless the sound pressure is strictly matched. Second, C57BL/6 J mice develop age-related hearing loss, making it unreasonable to use sound levels lower than 60 dB SPL. On the other hand, using very high sound levels such as 80 dB SPL in GCaMP imaging would distort the tonotopic map. Therefore, loud stimuli would not be appropriate in a study aiming to evaluate tonotopic/envelope map structure.

### 2.5 Cortical activities evoked by auditory stimulation across different ages

Age-related decline of peripheral auditory function was partially controlled for by confirming stable cortical amplitudes across different ages. For this purpose, response amplitudes (ΔF/F0) were measured in the right AAF and AI evoked by auditory stimulation at 5, 20, and 40 kHz. In this study, auditory brainstem recording, assessing the age-related decline of peripheral auditory function, was not performed but was substituted by measuring the above-mentioned cortical activities (ΔF/F0).

### 2.6 Tonotopic map

**Figure 2:**
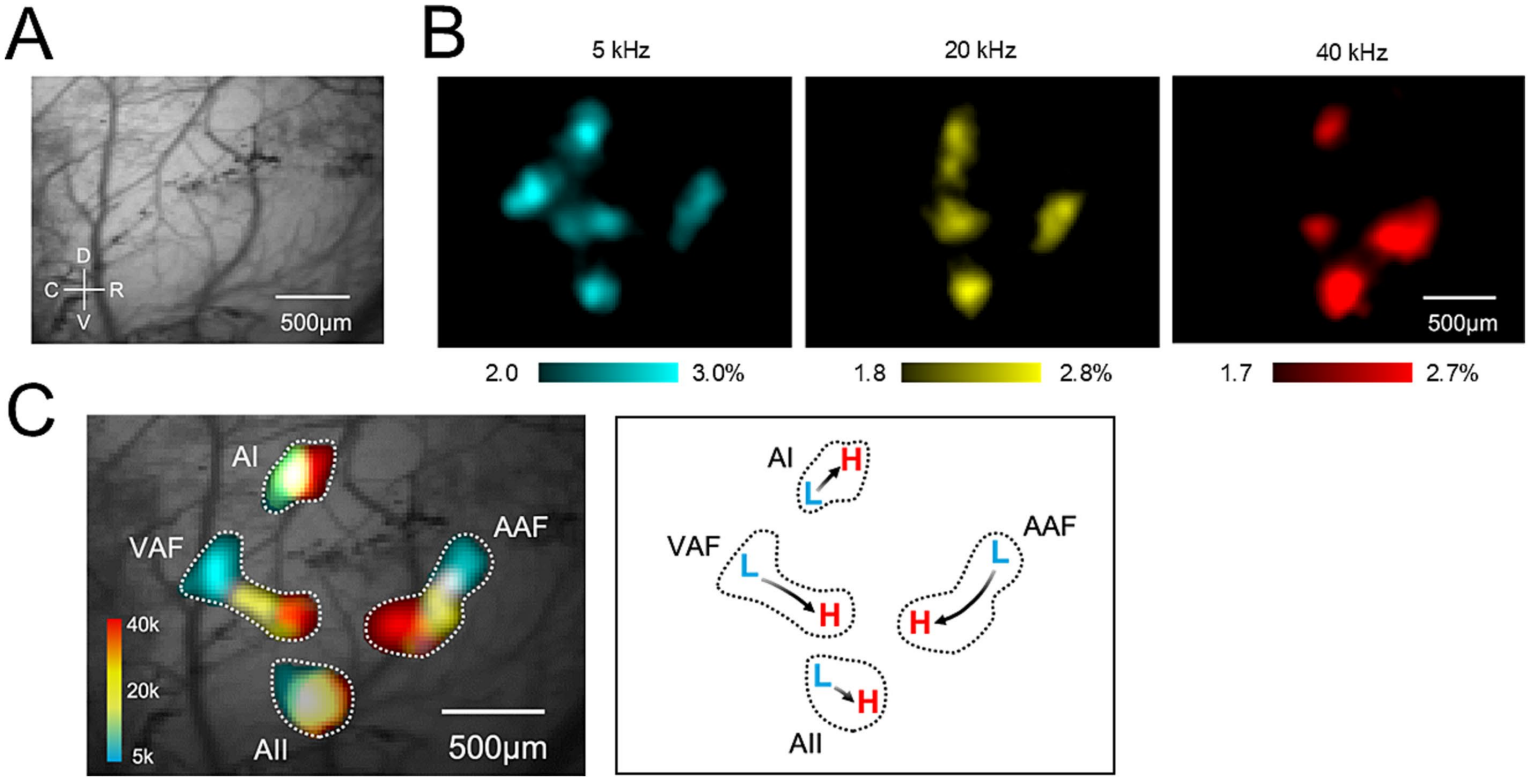
Right auditory cortex imaging (tonotopic map). **(A)** Example field view of the right auditory cortex. The brain surface was transcranially observed. C, caudal; D, dorsal; R, rostral; V, ventral. **(B)** Example images of peak responses to 5–40 kHz tones with a 100 ms rise-ramp in the auditory cortices. Each frequency elicited a response in different locations of the auditory cortices. **(C)** Tonotopic map of the right auditory cortex. Clear tonotopic gradients from low (L) to high (H) frequencies were observed in the AAF, VAF, AI, and AII. AAF, anterior auditory field; AI, primary auditory field; AII, secondary auditory field; VAF, ventral auditory field.

To visualize tonotopic maps, the mice were exposed to tones with frequencies ranging from 5 to 40 kHz and a rise-ramp time of 100 ms. Figure 2A shows a field-of-view of the right auditory cortex. To present representative responses for each frequency in Figure 2B, the response images were color-coded and thresholded using RatioImaging Analyzer (E.I.SOL). To generate the merged image shown in Figure 2C, responsive pixels were extracted from each auditory area. Within each area, responses were normalized to the local peak, and threshold levels (70–85% of the maximum peak) were determined by visual inspection using RatioImaging Analyzer (E.I.SOL) to reveal the tonotopic organization best. Pixels within the responsive regions were identified using a flood-fill algorithm initiated from the seed points placed in the activated areas. The extracted peak response regions were color-coded and merged into a single image using ImageJ (NIH, Bethesda, MD, United States). Stability of tonotopic maps across ages was investigated by measuring the peak location from that evoked at AAF by 5 kHz auditory stimulation.

### 2.7 Envelope steepness map and its age-related shift

**Figure 3:**
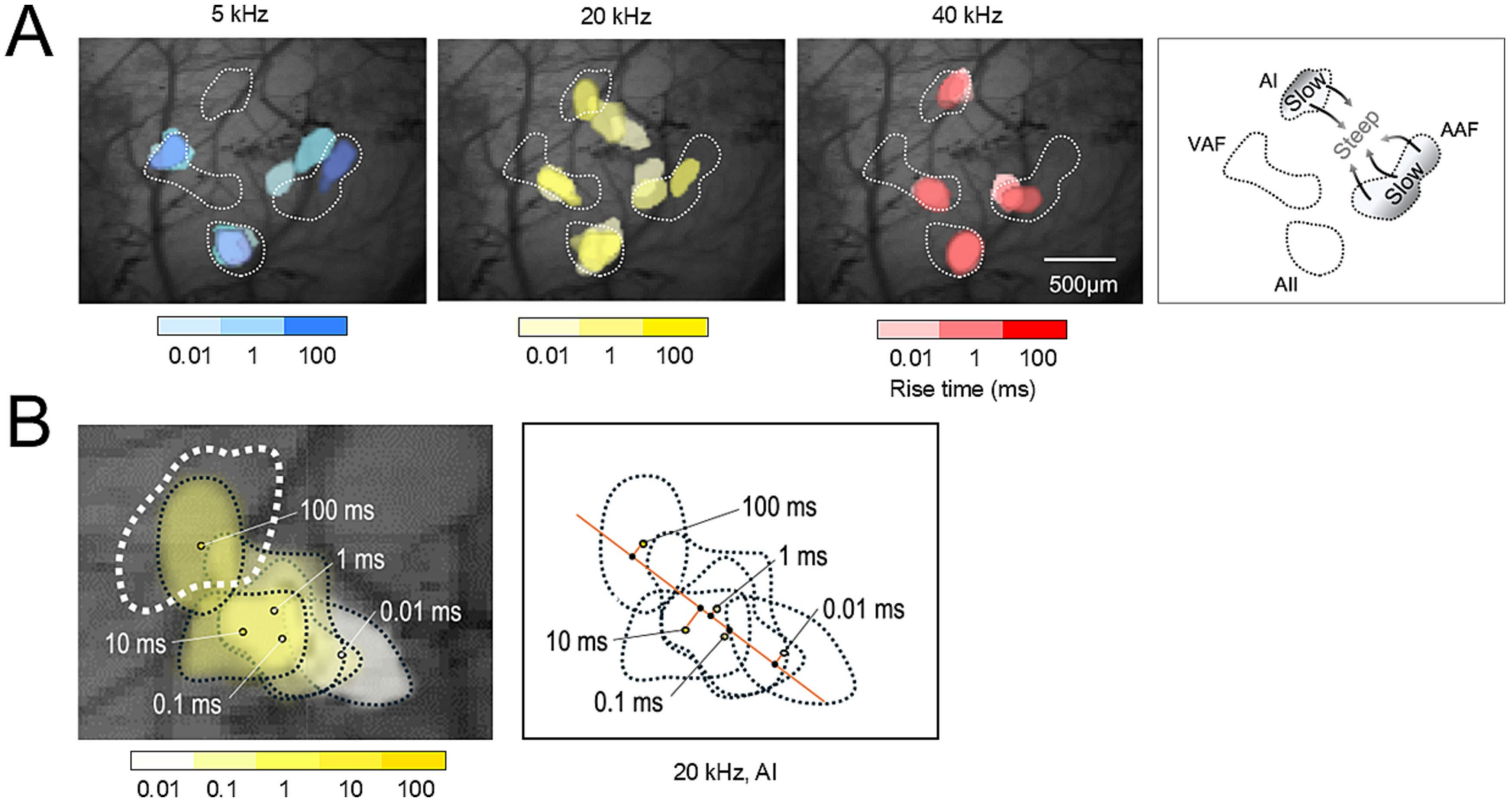
Right auditory cortex imaging (envelope steepness map). **(A)** The envelope steepness map visualized by superimposing threshold peak responses to a 5–40 kHz tone with rise-ramp times from 0.01 to 100 ms. The color gradation from light to dark represents a rise-ramp from short (steep) to long (slow) time. White dotted lines indicate the boundaries of the tonotopic maps. The direction of the rise-ramp gradients from slow to steep was orthogonal to the tonotopic gradients. **(B)** Magnified view of **(A)**: envelope steepness map in the AI responding to a 20 kHz tone. The white dotted lines indicate the boundaries of the tonotopic map, and the black dotted lines indicate the area corresponding to each rise-ramp time. The amber line is the fitted line of the response centers with different rise-ramp times, which is orthogonal to the direction of the tonotopic gradients in the AI (Figure 2C).

To present the representative response for each rise-ramp in Figure 3A, response images were separately normalized for each auditory area and thresholded to extract peak response regions, yielding binary images. Threshold levels (70–85% of the maximum peak) were similarly chosen by visual inspection to visualize the envelope steepness organization optimally. Responsive regions were extracted using the same flood-fill algorithm and merged using ImageJ (NIH).

The peak locations for different rise-ramp times were tested in the AAF, VAF, AI, and AII. The peak location was not significantly changed by different rise-ramp times in VAF and AII, suggesting the absence of an envelope steepness map for these areas. Age-related shift was investigated only in AAF and AI. Although it may have been ideal to include negative areas as controls, we anticipated that in aged mice the peaks corresponding to different rise–ramp times would become even closer together, making it difficult to detect any meaningful shifts. Changes in envelope steepness map were assessed across different ages. Then, mice were grouped by age as follows and assessed for statistical testing: 1/3 months and 6/9/12 months after birth. Although there was some variability in the sample sizes by age, grouping the animals into young and aged groups allowed us to secure a sufficiently large number of samples, especially for aged animals. Then, the statistical power achieved in our analyses can be considered reliable. To evaluate the age-related shift in the peak location according to the rise-ramp time, we drew a perpendicular line from each actual response center to the approximated line, which assumes that all response centers lie on a straight axis. The intersection point on the approximated line was then defined as the position of each response center (Figure 3B, right). The distance from the point of rise time 0.01 ms to each fitted point was defined as the distance of each sound rise time (0.1, 1, 10, and 100 ms). The distance relative to the rise-ramp time of 0.01 ms was plotted for each rise-ramp time (0.1, 1, 10, and 100 ms) on a logarithmic scale graph. The steeper the slope of the fitted logarithmic regression lines, the greater the distance between the peaks of the rise-ramp time of 0.01 ms and 100 ms. The slope of this regression line was compared between the groups at 1/3 months and 6/9/12 months after birth.

### 2.8 Statistical analyses

Statistical analyses were performed using IBM SPSS Statistics version 31 for Windows (IBM Corp., Armonk, NY, United States). The data were used to construct a one-way analysis of covariance designed to test the relative effect of age on the dependent variables, controlling for the possible interaction with and confounding influence of rise-ramp time as a continuous predictor variable. *p*-values <0.05 were considered significant.

## 3 Results

**Figure 4:**
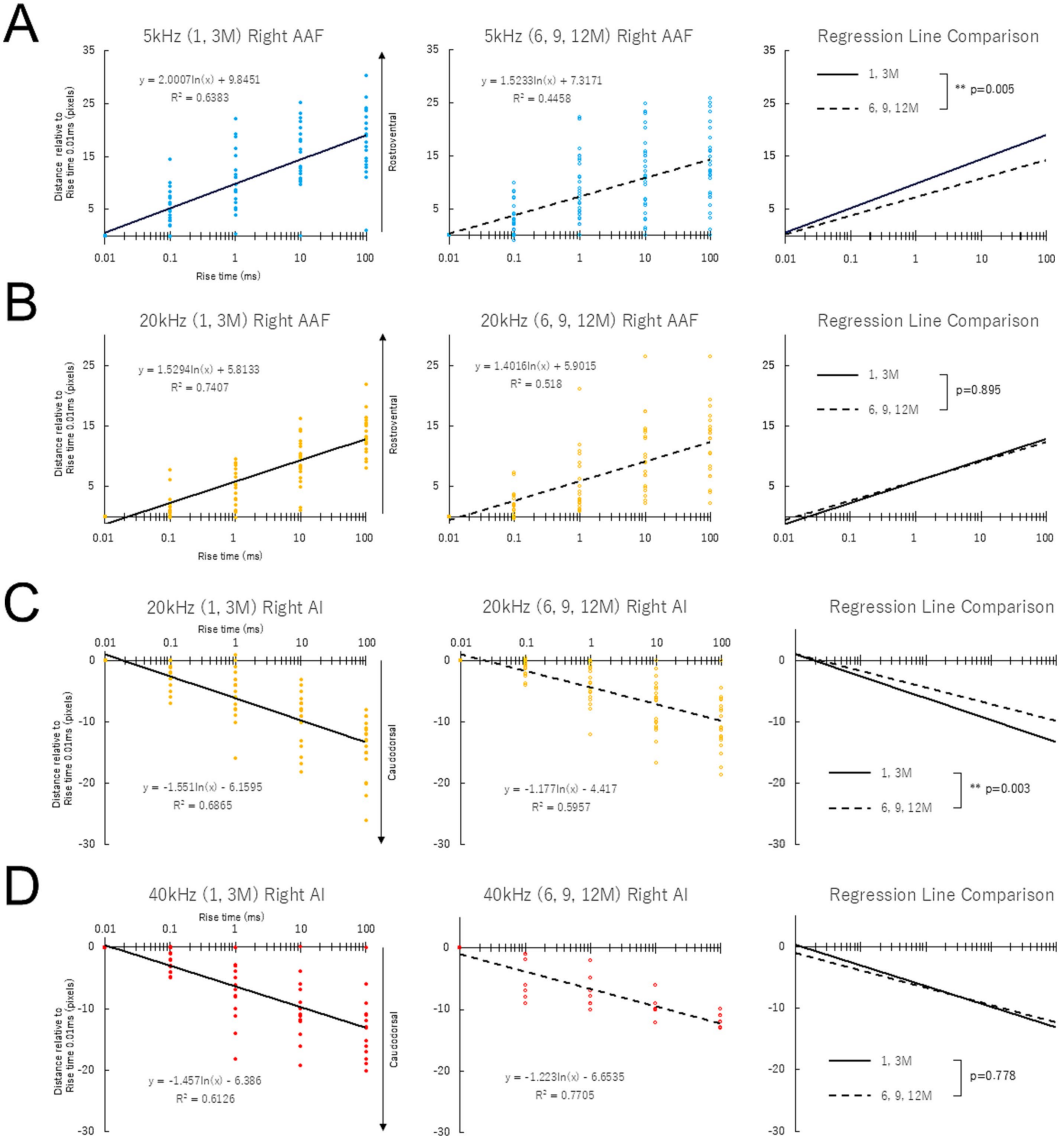
Response shift to rise time in the right AAF **(A,B)** and AI **(C,D)** in mice of different ages. The slope of the best-fit logarithmic regression line for 5 kHz and 20 kHz was significantly less steep in mice at 6, 9, and 12 months after birth than at 1 and 3 months after birth in the right AAF **(A)** and AI **(C)**, respectively.

**Figure 5:**
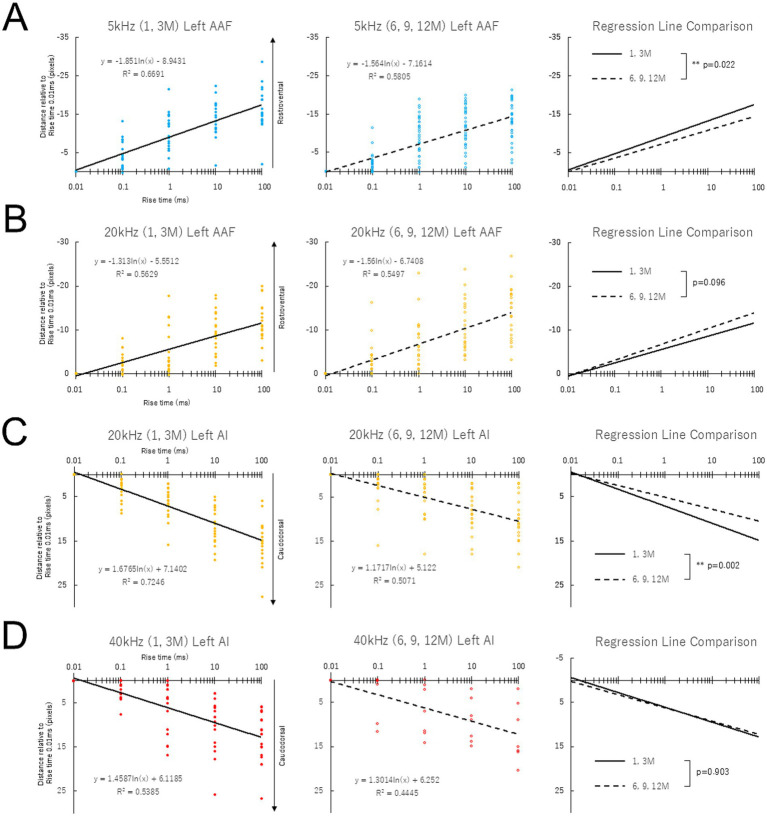
Response shift to rise time in the left AAF **(A,B)** and AI **(C,D)** in mice of different ages. The slope of the best-fit logarithmic regression line for 5 kHz and 20 kHz was significantly less steep in mice at 6, 9, and 12 months after birth than at 1 and 3 months after birth in the left AAF **(A)** and AI **(C)**, respectively.

Supplementary Table 1 illustrates the number of mice of different ages showing responses to each tone (5, 20, and 40 kHz) in the AAF and AI. In the AAF, aged mice tended to lose their response to high-frequency tones. In the AI, similar trends were shown as in the AAF; however, the response to a 5 kHz tone was sometimes not found, even in the youngest mice, 1 month after birth. Based on these results, an envelope steepness map was constructed for tones with 5/20 kHz in the AAF and 20/40 kHz in the AI (Figures 4, 5). Given the possible effects of age-related peripheral hearing loss on the auditory cortices, response amplitudes (ΔF/F0) were measured in the right AAF and AI evoked by auditory stimulation at 5, 20, and 40 kHz. While the response amplitudes for 20 and 40 kHz pure tone showed age-dependent decline in AAF and AI (Supplementary Figures 1-2, 1-3), those for 5 kHz pure tone were stable up to 12 months of age in AAF and AI (Supplementary Figure 1-1), suggesting that the cortical activity in response to 5 kHz pure tone was not affected by age.

Figure 2 illustrates an image of the right auditory cortex, demonstrating a tonotopic map. Figure 2A shows a field-of-view of the right auditory cortex. The brain surface was transcranially observed. Figure 2B shows example images of peak responses to 5–40-kHz tones with a 100-ms rise-ramp in the auditory cortices. Each frequency elicited responses in different locations of the auditory cortices. Figure 2C illustrates the tonotopic map of the right auditory cortex, which was visualized by superimposing the threshold response images shown in Figure 2B. Clear tonotopic gradients were observed in the AAF, VAF, AI, and AII (Figure 2C). The directions of the tonotopic gradients from low (L) to high (H) frequencies were consistent with those of previous studies (Narayanan et al., 2022; Tsukano et al., 2016; Liu et al., 2019). We also analyzed the stability of tonotopic maps across different ages. Supplementary Figure 2-1 shows the representative tonotopic map of each age in the right auditory cortex, demonstrating that the tonotopy was stable up to 12 months. Supplementary Figures 2-2–2-7 show the peak location of each age in the right (Supplementary Figures 2-2–2-4) and left (Supplementary Figures 2-5–2-7) auditory cortex as expressed by distance from the peak response to 5 kHz auditory stimulation in AAF. Peak location was not different among ages, also suggesting the stable tonotopy up to 12 months.

Figure 3 illustrates an image of the right auditory cortex, demonstrating an envelope steepness map. Figure 3A shows the envelope steepness map visualized by superimposing threshold peak responses to a 5–40 kHz tone with rise-ramp times from 0.01 to 100 ms. The color gradation from light to dark represents a rise-ramp from short (steep) to long (slow) time. The white dotted lines in Figure 3A indicate the boundaries of the tonotopic maps in Figure 2C. Figure 3B shows a magnified view of Figure 3A (20 kHz), illustrating the responses to a 20 kHz tone with different rise-ramp times in the right AI. The black dotted lines in Figure 3B indicate the corresponding areas for each rise-ramp time. The amber line is the fitted line of the response centers with different rise-ramp times (Figure 3B), which is almost orthogonal to the direction of the tonotopic gradients in the AI (Figure 2C), indicating the presence of two-dimensional maps in the right AI that represent tonotopy and envelope steepness.

Supplementary Figures 3-1–3-3 show example data on changes in envelope steepness map across different ages, showing stable maps up to 12 months in the right auditory cortex. Data were obtained from the same animals used for assessing age-related changes in the tonotopic maps. Figure 4 shows the response shift to rise time in the right AAF (A, B) and AI (C, D) in mice of different ages. Normalized distance relative to the rise-ramp time of 0.01 ms was plotted on a logarithmic scale. The steeper the slope of the fitted logarithmic regression lines, the greater the distance between the peaks of the rise-ramp time of 0.01 ms and 100 ms. The slope of the best-fit logarithmic regression line for 5 kHz and 20 kHz was significantly less steep in mice at 6/9/12 months after birth than at 1/3 months after birth in the right AAF (Figure 4A) and right AI (Figure 4C), respectively. Supplementary Figure 4-1 presents the slope of the regression line of the envelope steepness map for each age group before pooling each data, demonstrating gradual decline in response to sound stimulation with 5 kHz in AAF and 20 kHz in AI. Information about the effect size, mean and SEM was provided as Supplementary Figure 4-2: Cohen’ d was 0.533 and 0.826 for 5 kHz AAF and 20 kHz AI, respectively, suggesting moderate to large effects by age on the slope of regression lines.

Figure 5 shows the data corresponding to Figure 4 for the left AAF and AI. The same findings were obtained on the left (Figure 5) and right sides (Figure 4). As shown in Supplementary Figure 4-3, there were no left–right differences in the slope of the regression line in auditory cortices of young and aged animals.

## 4 Discussion

As shown in Figures 2, 3, the mouse auditory cortex contains well-defined two-dimensional maps, that is, a tonotopic map (Figure 2) and an envelope steepness map (Figure 3), where tonotopy and envelope steepness are orthogonally represented. These results are consistent with our previous data (Takahashi et al., 2025). Although the auditory cortex is critical for perceiving both temporal envelopes (Nourski et al., 2009) and pitch (frequency information) (Warrier and Zatorre, 2004), the orthogonal representation of envelope steepness and tonotopy suggests that temporal envelopes are processed independently of spectral features in the auditory cortex, supporting the psychological observation that the temporal envelope and carrier frequency work independently in verbal communications (Smith et al., 2002). The rise-ramp of the sound envelope may affect the perception of speech; sounds with a steep rise are easier to discriminate than those with a slow rise (Schlittenlacher and Ellermeier, 2015; Shimokura and Soeta, 2022). Therefore, the envelope steepness map may play a crucial role in the perception of speech rather than in discriminating the pitch of a pure tone, for which the tonotopic map works (Milne et al., 2016).

Studies using mouse models have explored the changes in the central auditory system following the onset of hearing loss and have reported qualitative changes in the arrangement of tonotopic maps in the auditory cortex (Yang et al., 2011; Persic et al., 2020; Resnik and Polley, 2021). Given the possible independent role of the envelope steepness map from the tonotopic map in auditory perception, it can be hypothesized that the accuracy of the envelope steepness map, that is, the separating ability of rise-ramp steepness, may play an important role in speech recognition and its age-related decline. Certainly, as shown in Figures 4, 5, the slope of the best-fitted logarithmic regression line plotting the distance of the peak location versus rise-ramp time for 5 kHz in bilateral AAF and 20 kHz in bilateral AI was significantly less steep in animals at 6/9/12 months after birth than in animals at 1/3 months after birth, suggesting that aged animals may have difficulty in separating the rise-ramp time. These findings may support the cortical mechanisms underlying age-related decline in speech perception.

The degeneration of the envelope steepness map is clearly an age-dependent change. However, even during the mid-aging stage (6–12 months), when overt hearing loss has not yet occurred, we could observe degeneration of the envelope steepness map at 5 kHz. This finding suggests that alterations in temporal processing may occur prior to the onset of peripheral hearing loss. If this interpretation is correct, it is possible that degeneration in the envelope steepness map corresponding to higher frequency ranges—where age-related hearing loss progresses relatively early—may have already begun in the young group. Indeed, even within the young group, we observed a tendency for the slope of the map to gradually decrease as frequency increased (Figures 4, 5). Thus, in the higher frequency ranges, differences between the young and aged groups may not have been clearly detectable. At 20 kHz in AAF (Figures 4, 5), the observed intermediate phenotype may reflect a process in which degeneration had already begun during the young stage. Further investigation will be required to elucidate the detailed mechanisms underlying these changes.

In human speech recognition, temporal changes in sound, particularly the speed of sound rise, are considered fundamental (Rosen, 1992; Shannon et al., 1995; Ríos-López et al., 2017). Rapid temporal dynamics are essential for distinguishing speech sounds, perceiving syllables, and decoding complex auditory cues, such as rhythm and pitch (Schlittenlacher and Ellermeier, 2015; Shimokura and Soeta, 2022). Moreover, even when pure-tone audiometry, which measures basic hearing thresholds, is normal, older adults may experience challenges processing temporal information (Eisenberg et al., 1995; Peters et al., 1998; George et al., 2006; Summers and Molis, 2004). This highlights the crucial role of the central auditory system in the temporal processing, which extends beyond the sensitivity of peripheral auditory receptors (Moore et al., 1992; Noordhoek et al., 2001; Bernstein et al., 2013). It is indeed true that directly relating the present findings—obtained from mice that likely exhibit peripheral hearing loss—to age-related hearing loss in humans, where speech perception declines even in the absence of significant peripheral impairment, may not be appropriate. However, results of this study would suggest that mice exhibit an age-related decline in temporal processing. This decline in auditory processing may underlie broader difficulties in distinguishing subtle variations in sounds with age. Individuals with impaired speech perception may also experience a deterioration in the functional organization responsible for representing temporal features. Understanding these mechanisms in animal models may provide insights into the biological basis of auditory aging and speech perception challenges in humans.

In this study, cochlear peripheral function was not controlled between young and old animals, and the decline in cochlear function associated with aging might also contribute to the decline in perception of envelope steepness and speech. Indeed, the response amplitudes for 20 and 40 kHz pure tone showed age-dependent decline in AAF and AI (Supplementary Figure 1-3). However, those for 5 kHz pure tone were stable up to 12 months of age in AAF and AI (Supplementary Figure 1-1), suggesting that the cortical activity in response to 5 kHz pure tone was not affected by age. Importantly, degeneration of the envelope steepness maps due to aging, i.e., decreased slope of the regression line plotting rise-ramp time vs. normalized distance (Figure 4A), was even observed in the frequency range in which no effects of aging-related hearing loss were detected (5 kHz). This strongly suggests that degeneration of the envelope steepness maps may occur independently of age-related peripheral hearing loss.

As another possibility of age-related degeneration of the envelope steepness maps, indirect effects via changes in tonotopic maps may be rejected. As shown in Supplementary Figures 2-1–2-7, the peak location of all tonotopic maps in the auditory cortices of both sides was stable across different ages. These findings suggest that temporal envelopes and spectral tonotopic features are independently processed in the auditory cortex and that age-related degeneration is specifically seen in the temporal processing.

In conclusion, the mouse auditory cortex contains well-defined two-dimensional maps, that is, tonotopic and envelope steepness maps, in which tonotopy and envelope steepness are orthogonally represented. The distance of the peak location for different rise-ramp times in the auditory cortex was short in aged mice, supporting cortical mechanisms contributing to age-related declines in speech perception.

## Data Availability

The raw data supporting the conclusions of this article will be made available by the authors, without undue reservation.
